# NEONATAL PEMPHIGUS IN AN INFANT BORN TO A MOTHER WITH PEMPHIGUS VULGARIS: A CASE REPORT

**DOI:** 10.1590/1984-0462/;2019;37;1;00004

**Published:** 2018-07-26

**Authors:** Adriana Amaral Carvalho, Dinamar Amador dos Santos, Mirelle Augusta dos Reis Carvalho, Sabrina Jeane Prates Eleutério, Alessandra Rejane Ericsson de Oliveira Xavier

**Affiliations:** a Universidade Estadual de Montes Claros, Montes Claros, MG, Brasil.; b Hospital Municipal de Paracatu, Paracatu, MG, Brasil.

**Keywords:** Pemphigus, Autoimmune diseases, Newborn infant, Pênfigo vulgar, Doenças autoimunes, Recém-nascido

## Abstract

**Objective:**

To report on the case of a patient with neonatal pemphigus that had extensive and critical skin lesions at birth.

**Case description:**

A newborn male with extensive vesico-bullous lesions on the anterior side of his chest and abdomen at birth. He was admitted to the pediatric ward of a hospital for an etiological diagnosis and for treatment. Based on maternal history and a clinical evaluation, the patient was diagnosed with neonatal vulgar pemphigus. His progression was satisfactory and, in the end, he did not need pharmacological interventions.

**Comments:**

The cases reported in the literature and the references evaluated reveal that neonatal pemphigus is rare, but that knowledge about the disease allows for an early diagnosis to be made. This has great clinical relevance considering that the disease usually manifests itself in the form of extensive epidermal lesions, even though it is transient and benign, it does not require specific treatment, and it does not have any relation with possible future diseases.

## RESUMO

**Objetivo:**

Relatar um caso de pênfigo neonatal em paciente que manifestou lesões cutâneas extensas e críticas ao nascimento.

**Descrição do caso:**

Recém-nascido do sexo masculino com lesões vesicobolhosas extensas em região anterior do tórax e abdome, desde o nascimento. Admitido na ala pediátrica de um hospital para diagnóstico etiológico e tratamento. Com base na história materna e na avaliação clínica, concluiu tratar-se de pênfigo vulgar neonatal. O paciente apresentou evolução satisfatória, sem a necessidade de intervenção farmacológica.

**Comentários:**

Os casos descritos na literatura e as referências avaliadas revelam o pênfigo neonatal como uma doença de ocorrência rara, porém cujo conhecimento e diagnóstico precoce têm grande relevância clínica, considerando-se que geralmente se manifesta com lesões epidérmicas extensas e de aspecto crítico, embora apresente curso clínico transitório e benigno, sem necessidade de tratamento específico e sem relação com doença futura.

## INTRODUCTION

Pemphigus vulgaris is a disease that is characterized by flaccid blisters and erosions, which are caused by the presence of autoantibodies that act against epidermal components, such as desmogleins (DSG).^1^ ^,^ ^2^ ^,^ ^3^ ^,^ ^4^ ^,^ ^5^ ^,^ ^6^ It is uncommon in the pediatric population, representing about 1.4 to 2.9% of all cases.^1^ ^,^ ^7^ With regard to its clinical manifestations, pemphigus vulgaris usually begins with superficial and ephemeral bubbles in the oral mucosa. These manifestations go into remission and reoccur over a period of months, until there are skin blisters interspersed with healthy skin, which is prone to generalization.^8^ A diagnosis is made based on the association of the clinical findings with the result from a biopsy of the affected skin and from the immunofluorescence or enzyme-linked immunosorbent assay (ELISA),^8^ which demonstrates the intraepidermal deposition of type 1 and 4 immunoglobulins (IgG).^4^ Pemphigus vulgaris treatment is based on the use of high doses of corticosteroids,^1^ ^,^ ^2^ ^,^ ^3^ ^,^ ^4^ especially prednisone, and it is sometimes necessary to add on immunosuppressants such as methotrexate, cyclophosphamide, mofetil mycophenolate, and azathioprine.^8^ Progression is chronic, and a refractory disease in response to the treatment often occurs. The use of rituximab in such cases has shown to be promising.^8^

Neonatal pemphigus is an autoimmunce blistering disease caused by the transfer of maternal IgG (representing autoantibodies against desmoglein-3), through the placenta, when the mother is affected by pemphigus.^5^ It is expressed in children of pemphigus carriers after the occurrence of a transient event, which stops the maternal antibodies from disappearing. The clinical manifestation of neonatal pemphigus is less severe in comparison to the disease that caused it, as it is not a systemic disease. The signs and symptoms of neonatal pemphigus are restricted to skin lesions, and they have a good prognosis. It is expected for symptoms to be resolved within three weeks.^5^

In this article we describe the case of a patient with neonatal pemphigus vulgaris, who progressed satisfactorily, demonstrating the clinical behavior expected without the use of immunosuppressants or antibiotics.

## CASE REPORT

A newborn patient, from the municipality of Mirabela, Minas Gerais, was admitted to a hospital in Montes Claros, Minas Gerais on his second day of life. The patient was male, black with light-colored skin, and was born from a cesarean section (because of functional dystocia). At birth, he was full of life, and had an Apgar score of 9 and 10 after 1 and 5 minutes, respectively. He was born after a 40-week pregnancy and weighed 3,250g at birth.

The mother was 37 years old, had had three pregnancies and had never performed an abortion. She attended nine pre-natal consultations, and her test results showed negative serologies for congenital infections. She had a history of urinary tract infection in the third trimester of pregnancy, and was treated with a cure control. Before the current pregnancy, the mother had lesions on her skin and underarms. After performing a biopsy of these tissues, the histological results revealed findings compatible with pemphigus vulgaris. The mother was therefore diagnosed with pemphigus vulgaris a few months after the beginning of the previous pregnancy. Since then, she has followed up with a dermatologist. She used azathioprine up until the first month of the current pregnancy (she stopped when the pregnancy was confirmed), and prednisone during the whole pregnancy period. The disease became active again in the second trimester of the current pregnancy, however the lesions went into remission for a short period after increasing the dose of corticoid.

**Figure 1:**
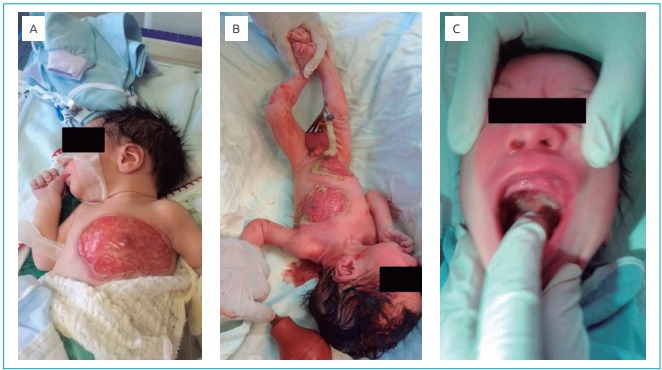
Lesions that are characteristic of neonatal pemphigus vulgaris at birth. (A and B) photographic images showing extensive vesicobolous lesions in the anterior region of the thorax and the abdomen of the newborn. The edges are well defined, have a scaly appearance and bullish vesicles with crusts permeating their interior; (C) lesions on the palate of the newborn.

The newborn was transferred in order to make a diagnosis and to treat the extensive vesicobolous lesions in the anterior region of the thorax and abdomen (Figures 1A and 1B), as well as the oral lesions (Figure 1C) present since his birth. When he was admitted, he was in good general condition - he was active and reactive, he had preserved capillary perfusion, he was eupneic in the ambient air, and he had no apparent malformations. The patient had a rounded lesion on his chest. The lesion had an erythematous base that was approximately 4 cm in diameter. It was well delimited and had scaly borders with vesicles, bubbles and crusts permeating the inside (Figures 1A and 1B). On his abdomen, the patient had an extensive lesion with similar characteristics, measuring 6 × 4 cm (Figures 1A and 1B). Laboratory tests were performed as normal to screen for infectious diseases.

**Figure 2:**
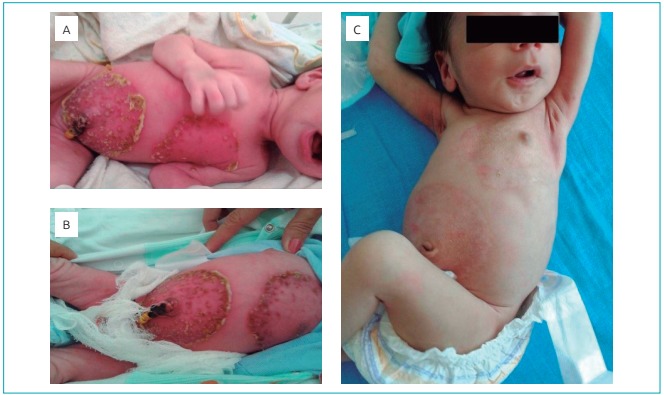
The progressive remission process of lesions that are characteristic of pemphigus vulgaris in the newborn. (A, B and C) photographic images showing an improvement in the extensive vesicobolous lesions in the anterior region of the thorax and the abdomen of the newborn.

Based on the mother’s medical history, the patient’s lesions, and an evaluation of clinical and laboratory evidence, the diagnostic hypothesis was neonatal pemphigus. The child progressed favorably without the use of medication, the lesions went into remission, and there were no signs of a secondary infection (Figures 2A to 2C). The dermatological team evaluated the situation and considered it unnecessary to perform a cutaneous biopsy, considering the epidemiological history and the clinical course of the disease, which was consistent with the diagnosis of neonatal pemphigus vulgaris.

The patient was discharged on the 16^th^ day of his life, because the extensive lesions had improved greatly. He returned and was evaluated at the pediatric outpatient clinic of the hospital on his 23^rd^ day of life, where he was in complete remission of the condition (Figure 2C).

This study was approved by the Research Ethics Committee (CEP) of the Universidade Estadual de Montes Claros (Unimontes) (Report number 2.112.276 / 2017). The data were collected only after the mother of the newborn had signed a free and informed consent form.

## DISCUSSION

Pemphigus vulgaris occurs rarely in pregnancy,^9^ however it is important to know about the disease due to its impact on the mother’s pregnancy and on the fetus. For these reasons, management is always a challenge. Ideally, preventive measures should be adopted even before conception. As with other autoimmune diseases, pemphigus may be associated with the difficulty to conceive.^2^ In addition, certain behaviors should be adopted to prevent some types of immunosuppressive agents, and the dose of corticosteroid taken must be reduced to the lowest possible effective dose in order to avoid complications, if pregnancy occurs.^2^

In the case of the pregnant woman presented in this report, the use of azathioprine was suspended only after the first month of pregnancy, when the pregnancy was discovered. This imposed additional risks for the fetus. It is preferable to have adequate control over the disease prior to pregnancy, since biliary autoimmune diseases can be altered by a pregnancy, presumably due to hormonal influences. The disease can begin or can be exacerbated as the pregnancy progresses and immediately after delivery.^10^ ^,^ ^11^ Lehman et al*.*,^12^ however, relate the adverse course of the disease during pregnancy to poor control as there are high titers of pemphigus antibodies. Studies on the genesis of autoimmunity in pemphigus vulgaris suggest the association with certain HLA (human leucocyte antigen) genotypes in Brazil, especially DR and DQ loci, which generate B cells that are responsible for the production of specific autoantibodies.^13^ Self-reactive T helper lymphocytes that recognize the extracellular domain of the desmogleins appear to be critical in the pathogenesis of the disease, regulating the activation of circulating B lymphocytes.^14^

In the case described, the disease became active again in the mother during the second trimester of pregnancy, which is in accordance with the literature. According to Weinberg,^15^ disease control improves in the third trimester of pregnancy, which can be explained by the increase in the placenta’s endogenous production of corticosteroids and its consequent immunosuppression.^2^ ^,^ ^10^

Neonatal pemphigus occurs in 30% to 45% of carriers’ children due to the transfer of maternal antibodies from the placenta to the fetus.^2^ Although it is transient, has a benign progression, and is unrelated to the development of future diseases, pemphigus in a newborn child - which sometimes has extensive and critical lesions, as in the presented patient - leads to different early and accurate diagnoses. This occurs because several other dermatoses may be present in the neonatal period, such as vesicles, pustules, blisters, erosions and ulcerations, all of which differ considerably in etiology, distribution, evolution and treatment. Understanding the difference between a transient, non-infectious disease and a potentially serious disorder is essential in order to save lives through timely diagnoses and interventions.^16^ ^,^ ^17^

When a diagnosis of neonatal pemphigus is confirmed, the treatment acts as a support, and the lesions are expected to regress after three weeks. However, a variable therapeutic approach has been described in the literature, which includes expectant behavior management and/or the use of systemic corticosteroids, which may or may not be associated with antibiotic therapy. The use of corticosteroids, the main treatment option for children and adults with pemphigus, is not supported by the neonatal pemphigus approach, because of its potent anti-inflammatory and immunosuppressive effect.^18^ Characteristically, neonatal pemphigus is a transient disease that is secondary to the passive transmission of maternal antibodies through placenta.^13^ The empiric start of antibiotics has been described in the literature^4^ when infectious bullous lesions are suspected, which is an important difference in the diagnosis of pemphigus. For the patient here presented, the clinical behavior defined by strict clinical observation in a hospital environment, in addition the to taking care of the skin in order to avoid secondary infection, were effective.

It should be noted that although a biopsy officially determines the disease, in this case, it was not necessary due to the evidence obtained through rigorous collection of data and a satisfactory clinical progression during the period of the patient’s clinical hospital observation.
